# Seasonal and geographical impact on human resting periods

**DOI:** 10.1038/s41598-017-11125-z

**Published:** 2017-09-06

**Authors:** Daniel Monsivais, Kunal Bhattacharya, Asim Ghosh, Robin I. M. Dunbar, Kimmo Kaski

**Affiliations:** 1 Department of Computer Science, Aalto University School of Science, P.O. Box 15400, FI-00076 AALTO Espoo, Finland; 2 Department of Experimental Psychology, University of Oxford, South Parks Rd, Oxford, OX1 3UD United Kingdom

## Abstract

We study the influence of seasonally and geographically related daily dynamics of daylight and ambient temperature on human resting or sleeping patterns using mobile phone data of a large number of individuals. We observe two daily inactivity periods in the people’s aggregated mobile phone calling patterns and infer these to represent the resting times of the population. We find that the nocturnal resting period is strongly influenced by the length of daylight, and that its seasonal variation depends on the latitude, such that for people living in two different cities separated by eight latitudinal degrees, the difference in the resting periods of people between the summer and winter in southern cities is almost twice that in the northern cities. We also observe that the duration of the afternoon resting period is influenced by the temperature, and that there is a threshold from which this influence sets in. Finally, we observe that the yearly dynamics of the afternoon and nocturnal resting periods appear to be counterbalancing each other. This also lends support to the notion that the total daily resting time of people is more or less conserved across the year.

## Introduction

In modern societies, daily activities are marked by the entrainment of the human circadian clock, which is strongly influenced by two different cues, each one following a 24-hour period but with a different offset. The first cue is related to environmental and sun-based events, and therefore being subjected to seasonal variations due to the yearly movement of earth around the sun. The second cue follows a local civil time, where social and economical factors impose restrictions on the timing and routine of human activities^1^. Regardless of the nature of the external cue participating in the entrainment of the human circadian clock, any change in it has a direct influence on various aspects of human life^2, 3^.

For human circadian rhythms, particularly for the sleep wake cycle (SWC), there are various biological^4^, sociological^5^ and environmental^6^ cues affecting the entrainment of the circadian clock, sometimes with undesirable effects on the mental and physical health of individuals^2, 7–12^. Due to its importance, the SWC has been studied in recent years from different perspectives, trying to understand and identify what are the processes and pacemakers governing its dynamics^13, 14^. Broadly speaking the current understanding of the generation and maintenance of SWC is that sleep is governed by two main mechanisms consisting of the 24-hour circadian system and awake-dependent homeostatic build-up of sleep pressure. Then sleep itself feeds back to regulate the circadian system and homeostatic sleep pressure build-up. Light plays a key role in synchronising and pace-making the circadian cycle or rhythm, gated by the SWC in suppressing the melatonin production in the pineal gland, regulating alertness. Apart from these, the daily social activities also play a role in driving SWC.

In general, the current research on the human SWC has focused on experiments on small groups under controlled situations^8, 11^, and on studies based on questionnaires^6, 15, 16^, mainly the Munich Chronotype Questionnaire (MCTQ). The use of questionnaires for studying SWC are proved to be efficient when assessing sleep wake cycle, but have some limit to the domain of its applicability^17^. Also, it has been suggested that humans living in modern societies are subjected to new environments, like electrical lighting^18, 19^, and exposure to these has disrupted and changed people’s natural sleeping habits, although there is no agreement in the field about these possible changes^19–23^. Studies of the sleeping patterns of people living in pre-industrial societies^20^ have shown that their sleeping times are similar to those of modern societies, and that temperature could play an important role in the dynamics of sleep. In the recent past the presence of new communication technologies as well as the accessibility to large-scale techno-social datasets (‘Big data’) have allowed the study of human behavior from diverse perspectives applying reality mining techniques. In particular, mobile phone call detail records (CDRs) have been analysed to study mental health^24^, social networks^25–28^, sociobiology^29–33^, behavior of cities^34, 35^, as well as mobility^36–41^.

In the recent past, data collected from sensors and logs from mobile phone have been used to study circadian rhythms^29, 32, 38, 42–44^. Aledavood *et al*.^29^ studying mobile phone calling patterns together with data collected from questionnaires, described the characteristic time at which the peak of alertness of a small cohort of 24 students occurs. In another study, comparing various medium size (around 100, 1200, 700 and 2400 participants) datasets of mobile phone records and emails, Aledavood *et al*.^45^ showed each individual level has a distinctive, persistent daily communication pattern that strongly differs from the average pattern.

**Figure 1:**
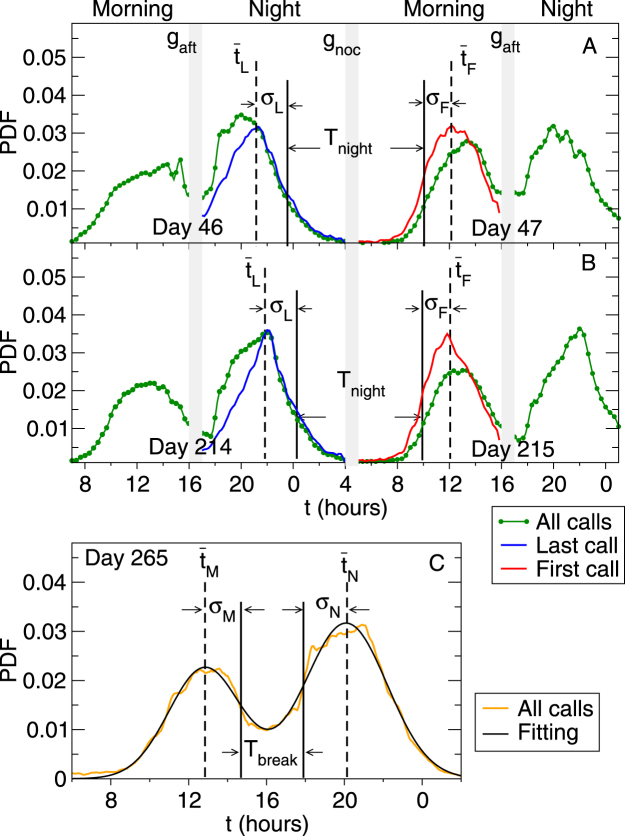
(Top panel) Probability distribution functions (PDF) of outgoing calls at time *t* of the day in a city for two sets of consecutive days of 2007: *P*_*all*_ when all the outgoing calls are included (Green), *P*_*L*_ when only the last outgoing calls in the *night* are included (Blue), and *P*_*F*_ when only the first outgoing calls in the *morning* are included (Red). From *P*_*L*_ and *P*_*F*_, their means, ${\overline{t}}_{L}$ and ${\overline{t}}_{F}$, and their standard deviations *σ*_*L*_ and *σ*_*F*_, respectively, are calculated and used to define the period of low calling activity (PLCA) as the region bounded by ${\overline{t}}_{L}$ and ${\overline{t}}_{F}$ and determine its width *T*_*night*_ as the time interval between ${\overline{t}}_{L}+{\sigma }_{L}$ and ${\overline{t}}_{F}-{\sigma }_{F}$. For the day 46 (middle of February, set A), *T*_*night*_ ≈ 10.5 hours, whilst for the day 214 (early August, set B), *T*_*night*_ ≈ 9.5 hours. (Note that *P*_*all*_ is delimited by the nocturnal calling gap *g*_*noc*_ (between 4:00 a.m. and 5:00 a.m.), *P*_*L*_ lies between the diurnal calling gap *g*_*aft*_ (from 4:00 p.m. to 5:00 p.m.) and *g*_*noc*_, whilst *P*_*F*_ is bounded by *g*_*noc*_ and *g*_*aft*_). (Bottom panel) Probability distribution of outgoing calls during the day 265 (Sunday, orange line) in a city (of label 2), fitted by a superposition of two normal (Gaussian) distributions (black line), the first centered round noon and the second one in the evening (8 p.m.). The fitting is done with the mean values *t*_*M*_ and *t*_*N*_, and the standard deviations *σ*_*M*_ and *σ*_N_, corresponding to the noon and evening-centered activity modes, respectively (see details in the text). The afternoon break period *T*_*break*_ is defined as $({t}_{N}-{\sigma }_{N})-({t}_{M}+{\sigma }_{M})$.

Users of the mobile phone network have specific time intervals when their calling activity ceases and it is expected that these periods are characterised by users taking rest. Each day shows two periods of low calling activity, the first after lunch time around 4:00 p.m., and the second coinciding with the night period, centered around 4:00 a.m. In turn, these two periods delimit two regions of high activity, one peaking around noon and the second one around 8:00 p.m. (see Fig. 1B). The daily calling activity described by these four periods follows a complex dynamics across the year and along different geographical zones, allowing it to be used to provide insight into the dynamics of human resting or sleeping pattern.

## Results

We analyse a dataset containing anonymised CDRs corresponding to a 12 month period in 2007 from a mobile phone service provider having subscribers in a number of cities in a country located in the Southern Europe subregion of the United Nations geoscheme^46^. The dataset contains more than 3 billion calls between 50 million unique identifiers, from which 10 million were associated with individuals having a contract with the company in question. The remaining identifiers belong to the subscribers of other companies or land-lines. Each call in the dataset involves at least one subscriber. For the majority of the subscribers, the age, gender, postal code, and location of the most accessed cell tower (MACT) are available, and we include into the analysis only those subscribers (termed as ‘users’ from here on) whose demographic information is complete. A user is considered to “live in a city” if the following three geographical locations – the associated city centre, the location of the MACT and the centre of the postal code are sufficiently close (details in SI). We choose cities having more than a hundred thousand inhabitants in the year 2007, such that our final analysis takes into account a set of 36 cities with around 1 million users in total.

The calling activity of an entire city depends on a number of factors, but it can be described in terms of two variables: the time of the day, and the date of the year. From the dataset, we calculate the probability distribution *p*_*all*_(*t*, *d*) of finding an outgoing call at time *t* of a day *d* = 1,…, 365 by a caller living in a particular city. We define a ‘day’ starting from 4:00 a.m. of a calendar day and running to 3:59 a.m. of the next calendar day. The reason of this choice is to clearly distinguish between different daily calling activity periods, separating them into two parts: those events occurring before the observed termination of the calling activity during the night and those occurring after that. The time when the calling activity falls to its minimal observed value in general occurs around 4:00 a.m., and we choose this natural bound as the beginning/end point of the calling activity ‘day’, delimiting the calling distributions *p*_*all*_ of different days.

In Fig. 1A we show *p*_*all*_(*t*, *d*) (green curve) during the two different pairs of consecutive days, *d* = 46–47 (marking mid-February) and *d* = 214–215 (the beginning of August) of 2007, for a city with around six hundred thousand inhabitants. The distribution *p*_*all*_(*t*, *d*) turns out to be bimodal with the first mode corresponding to the calls made during the morning, peaking around noon, and the second mode being related to calls during the evening, reaching its maximum around 8:00 p.m. Such bimodal patterns are found for all the days around the year and for all the cities included in this study. These two peaks are naturally delimited by two regions when the activity falls to a minimum, one between 4:00 p.m. and 5:00 p.m., associated mainly with the time after lunch, and a second at the end/beginning of the day, between 4:00 a.m. and 5:00 a.m., which lies inside the normal sleeping period. We will refer to the former as the afternoon calling activity minimum *g*_*aft*_ and to the latter as the nocturnal calling activity minimum *g*_*noc*_.

In order to study the periods of low activity, we split the day into two non-overlapping periods – ‘morning’ and ‘night’, each 11 hours long, delimited by *g*_*noc*_ and *g*_*aft*_. Here we define the ‘morning’ as the time period between 5:00 a.m. and 3:59 p.m., and ‘night’ the time period between 5:00 p.m. and 3:59 a.m. on the following calendar day. Each ‘morning’, a user can make a number of calls but to focus on the time when the calling activity starts we consider only the first call made during the ‘morning’ for each user and construct the associated probability distribution of the time of the first call *P*_*F*_(*t*, *d*). Similarly, we find the last call made by every user during the ‘night’ and construct the corresponding probability distribution for the time of the last call *P*_*L*_(*t*, *d*). It should be emphasized that in this study we include only the calls made by the users (outgoing calls), excluding all the incoming calls, because these may not depend on the activity pattern of the users. In Fig. 1A we compare the probability distribution for all the calls *P*_*all*_(*t*, *d*) with the corresponding distributions *P*_*L*_(*t*, *d*) and *P*_*F*_(*t*, *d*) for the times of the last call (blue) and first call (red), respectively, for two different pairs of consecutive days (during winter and summer) for the particular city with a population over six hundred thousand. The shapes of the distributions *P*_*L*_(*t*, *d*) and *P*_*F*_(*t*, *d*) depicted in Fig. 1A appear to be preserved for all the days and cities we have studied. For some particular days, mainly related to holidays and festivities, the corresponding data were filtered out from the analysis due to their atypical behavior (see Fig. A1 in SI).

From here on our assumption is that the two periods of low calling activity, characterise two commonly observed daily periods of low human activity (or resting periods). We identify the period of low calling activity occurring in the night, with the nightly sleep. There are results reported in the literature showing that analyses based on smart-phone usage describe the nocturnal sleeping period, thus giving support to our approach^42, 44^. In these studies as well as in ours, the underlying methods rely on estimating the sleep periods from the inactivity of the type of device from which the data is collected. Nonetheless, both methods have limitations on accurately estimating the sleep periods which vary depending on the nature of collected data.

For the case of the afternoon period of low calling activity, it has been shown in different studies^47–51^ that there is decay in the performance and intensity level of certain behavioral/physiological human activities, coinciding with the rising of the sleep propensity in the sleep/wake regulation. This period occurring during the afternoon has been associated with napping and/or resting^49–52^, and in general it is reported to occur between 3:00 p.m. and 6:00 p.m.^48, 49, 51^, which coincides with the afternoon period of low calling activity. As these periods overlap, we assume that the period of low calling activity is a good indicator of the resting time.

### Influence of latitude in seasonal variability of low-activity period

As regards to the calling activity presented in Fig. 1A, we expect the sleeping hours to be mainly concentrated in the night when the calling activity falls to a minimum. This nocturnal period of resting (NPR) is quantified in the following fashion. From distributions *P*_*L*_ and *P*_*F*_ for each city, we calculate the two means ${\overline{t}}_{L}(d)$ and ${\overline{t}}_{F}(d)$ to determine the times when the last call of the day and first call of the following morning were made, respectively. Thus the NPR is defined to be the region bounded by ${\overline{t}}_{L}(d)$ and ${\overline{t}}_{F}(d+\mathrm{1)}$. In order to estimate the duration of the period when the calling activity has practically ceased, we determine the width of NPR, *T*_*night*_, by taking into account the widths of the distributions *P*_*F*_ and *P*_*L*_, determined by the standard deviations *σ*_*F*_ and *σ*_L_, respectively. Hence, we can write ${T}_{night}(d)=24-({\overline{t}}_{L}\,(d)+{\sigma }_{L})+({\overline{t}}_{F}\,(d+\mathrm{1)}-{\sigma }_{F})$ (see Fig. 1A). This definition for the width of NPR does not require any introduction of arbitrary cut-off parameters.

**Figure 2:**
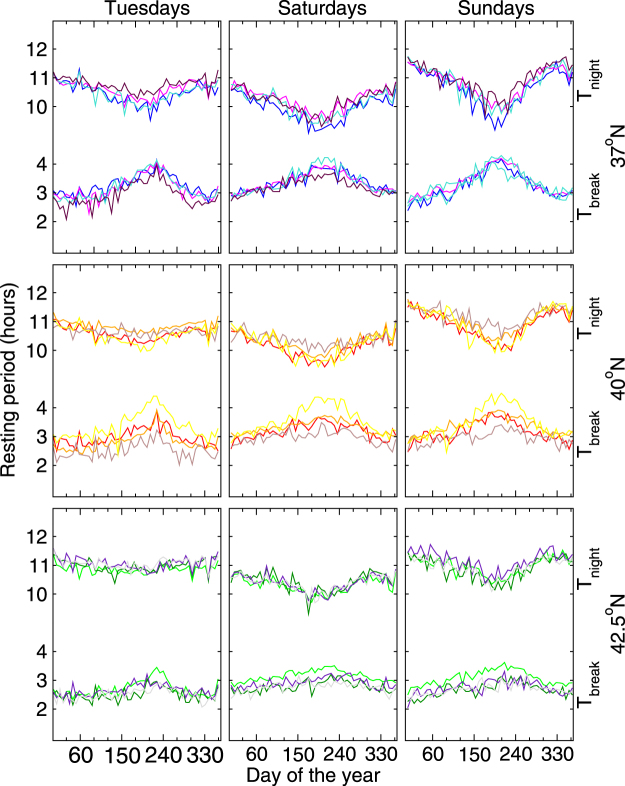
Periods of low calling activity or of resting: *T*_*break*_ and *T*_*night*_ for 12 different cities across 2007 for 3 different days of the week (Tuesdays, Saturdays, and Sundays in the left, central, and right column, respectively). Four cities are located in each one of the three different latitudinal bands (top) 37°N,(middle) 40°N, and (bottom) 42.5°N. For cities lying around 37°N, the color line associated with their times series are blue, magenta, turquoise and maroon; for cities at 40°N, the colors are red, orange, brown and yellow; and for cities around 42.5°N the colors are green, dark green, indigo and gray. Inside each one of the nine plots, the annual behavior of *T*_*break*_ (bottom lines) and *T*_*night*_ (upper lines) are shown, for the four different cities located at each band. On every plot, *T*_*break*_ and *T*_*night*_ show an opposite seasonal variations, with dynamics that appear to counterbalance each other, particularly on Sundays.

We have calculated *P*_*L*_ and *P*_*F*_ for 12 cities lying within one of three latitudinal bands, centered at 37°N, 40°N, and 42.5°N, in such a way that each band contains 4 cities. In Fig. 2 we show the yearly variation of *T*_*night*_ for these 12 cities. The plots show that the width *T*_*night*_ changes across the year, being longest near the winter solstice and shortest six months later, near the summer solstice. Comparing the different plots corresponding to *T*_*night*_, the following two characteristics should be noted. First, cities lying in the same latitude band show similar *T*_*night*_ curves, differing only by a vertical offset. This implies that although each city has a characteristic period of low activity, its yearly variation is very similar to that in other cities at the same latitude. Secondly, the difference between the highest (near winter solstice) and lowest values (near summer solstice) of *T*_*night*_ changes from latitude to latitude, being larger in southern cities and showing that there is an external factor that influences *T*_*night*_ with different intensity at different latitudes.

**Figure 3:**
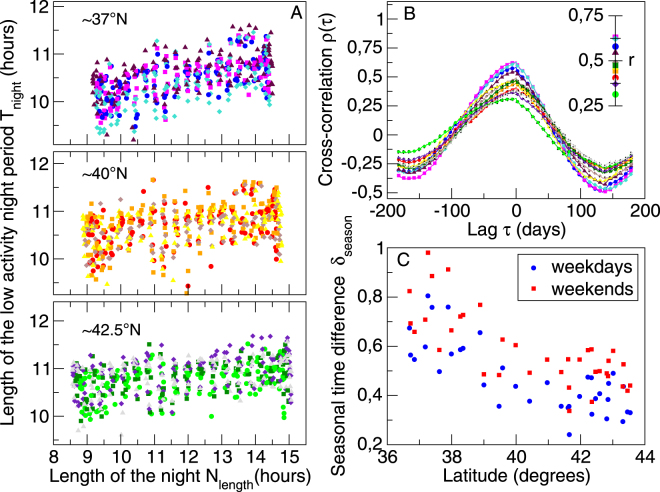
Relation between the period of low activity *T*_*night*_ and the length of the night *N*_*length*_: (Panel A) Scatter plots of *T*_*night*_ against *N*_*length*_ for 12 cities located at one of the three different latitudinal bands 37°N (blue, magenta, turquoise and maroon), 40°N (red, orange, brown and yellow), and 42.5N (green, dark green, indigo and gray), such that in each set the points represent the pairs (*N*_*length*_, *T*_*length*_) across the 365 days of 2007 (only the Tuesdays, Saturdays and Sundays are shown). (Panel B) The normalized cross-correlation *r* between *T*_*night*_ and *N*_*length*_ as a function of the time lag *τ*. Across the year *T*_*night*_ and *N*_*length*_ time-series are synchronized, as their cross-correlation *ρ*(*τ*) reaches its maximum for the time lag *τ* = 0, thus *T*_*night*_ and *N*_*length*_ are in phase and as depicted in the inset, the correlation between them is for all cases very high, i.e. *r* > 0.3, *p* < 0.001. The time-series were normalized (see Methods) and all the days of the week were taken into account. (Panel C) Latitudinal dependence of the net change *δ*_*season*_ between ${\overline{T}}_{night}$ at winter solstice (maximum) and at summer solstice (minimum). For cities in the south, this difference is larger than for the cities in the north. Each point corresponds to one of the 36 studied cities calculated as averages of ${\overline{T}}_{night}$ for weekdays from Mondays to Thursdays (blue) and for weekends from Fridays to Sundays (red).

As a matter of fact, the shape of *T*_*night*_(*d*) throughout the year resembles the yearly behavior of the length of the night *N*_*length*_(*d*), defined here as the time between the sunset and the sunrise for each city. The value of *N*_*length*_ peaks around the winter solstice (*d* = 356) and decreases monotonically until it reaches a minimum value around the summer solstice (*d* = 172), coinciding with the behavior of *T*_*night*_. In the scatter plots of ${T}_{night}$ vs. ${N}_{length}$ (Fig. 3A) we can see a noticeably linear dependence between these quantities, with the southern cities (≈37°N) showing the strongest effect. We use a linear regression, ${\overline{T}}_{night}=\beta {\overline{N}}_{length}+\alpha$, to quantify the yearly variation of ${\overline{T}}_{night}$ as a function of ${\overline{N}}_{length}$. Here ${\overline{T}}_{night}$ is defined as the average value of *T*_*night*_ from Monday to Thursday each week, to characterize typical weekdays and to reduce the fluctuations of *T*_*night*_. We carry out the regression for 36 different cities (including the aforementioned 12 cities) located between the 36°N and 44°N, for weekdays. Similarly, to characterize typical weekend days, we have calculated ${T}_{night}$ averaged across Fridays, Saturdays and Sundays. In order to study the effect of latitude on the seasonal variation of the NPR, we have calculated the net change *δ*_*season*_ between ${\overline{T}}_{night}$ for the winter solstice (maximum) and for the summer solstice (minimum), given by ${\delta }_{season}=\beta \cdot [{\bar{N}}_{length}(d=356)-{\bar{N}}_{length}(d=172)]$. This simple definition provides a way to calculate how the latitude affects the range where the period of low activity varies. For the 12 cities studied, we find that *N*_*length*_ and *T*_*night*_ are highly correlated, as can be seen in the inset in Fig. 3B, with the correlation *r* in the intervals [0.54, 062], [0.40, 048], and [0.31, 0.47], for the cities lying on the 37°N, 40°N, and 42.5°N latitudinal bands, respectively, with a confidence level greater than 99% in all cases. Besides this high correlation between *N*_*length*_ and *T*_*night*_, we explore if there is a time lag between their corresponding time-series. For this, we calculate the normalized cross-correlation *ρ* between the time-series for the same 12 cities as above (see Methods). In Fig. 3B it can be seen that *ρ* reaches its maximum when the time lag *τ* is zero, meaning that these time-series vary in the same phase. In Fig. 3C we show *δ*_*season*_ for each of the 36 cities at their own latitudes. Remarkably, the time difference *δ*_*season*_ between winter and summer is always at least 15 minutes larger for the southern cities compared to the northern cities. For weekends we observed a difference of 30 minutes, with almost a ratio 2:1 (30 minutes in the northernmost cities against one hour in the southernmost ones). The slopes of the linear regression obtained for each one of the 36 cities can be seen in the SI, Fig. A4. Cities around the same latitude have similar slopes (*β*s), and these slopes are larger for cities at the southern latitudes. In studies based on MCTQ, Allebrandt *et al*.^53^ and Kantermann *et al*.^54^ have reported that sleep duration variation across the year is around 20 minutes, for a sample of ≈55,000 people from Central Europe and a sample ≈58,00 people from Estonia, respectively. In contrast, in studies using wrist band activity sensors applied on subjects still living in hunter-gatherer societies (San and Tsimane), Yetish *et al*.^20^ reported that sleep duration of these tribes living in urban-free environments changes around 50 minutes across the year.

### Ambient temperature effects on the duration of diurnal resting period

For all the 36 cities included in this study and for almost every day, *P*_*all*_ shows apart from the nocturnal period of resting or predominantly night-time sleeping period, another low activity period centered around the diurnal gap *g*_*aft*_, ranging from 4:00 p.m. to 5:00 p.m. (Fig. 1C). The dynamics of this diurnal afternoon period of resting (DPR), associated with a decrease in human activity, can also be traced from the *P*_*all*_ dynamics though here the decrease is less dramatic than in the nocturnal case. We find that *P*_*all*_ can be described by the sum of two normal distributions, with one of the activity peaks centered around the noon, and the other in the evening around 8:00 p.m. as seen in Fig. 1C. Unlike in the NPR case where almost all the activity of people ceases due to them sleeping, the DPR shows a more moderate decrease in activity between these two peaks. This is associated with only a fraction of people having naps or taking rest. The length of this low activity period DPR can be measured with the fitting function given by $F(x)=\frac{{a}_{0}}{{\sigma }_{M}\sqrt{2\pi }}{e}^{-0.5{((t-{\bar{t}}_{M})/{\sigma }_{M})}^{2}}+\frac{{a}_{N}}{{\sigma }_{N}\sqrt{2\pi }}{e}^{-0.5{((t-{\bar{t}}_{N})/{\sigma }_{N})}^{2}}$. Here ${\overline{t}}_{M}$ and *σ*_*M*_ are the mean and the standard deviation of the noon-centered distribution and ${\bar{t}}_{N}$ and *σ*_N_ the corresponding values for the evening-centered one. Hence we define the duration of the DPR or the afternoon break period to be *T*_*break*_ as $({\overline{t}}_{N}-{\sigma }_{N})-({\overline{t}}_{M}+{\sigma }_{M})$ (Fig. 1C). The yearly change in *T*_*break*_ for 12 cities located at one of three different latitudinal bands 37°N, 40°N, or 42.5°N is depicted in Fig. 2.

**Figure 4:**
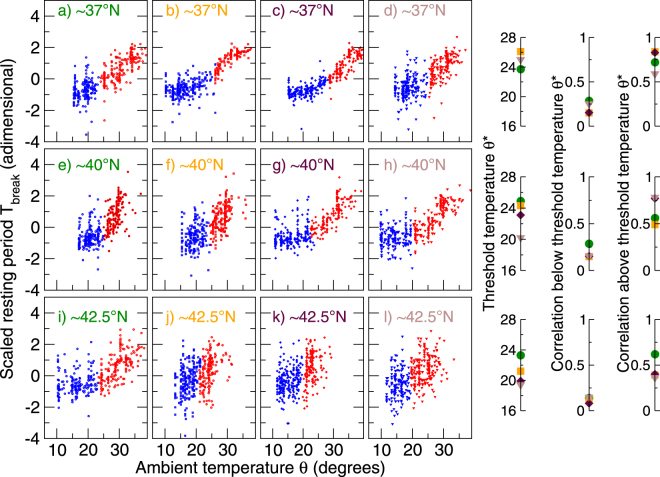
Relation between the afternoon break period *T*_*break*_ and the maximum daily temperature *θ*, for 12 different cities located at one of the three different latitudinal bands: (top) 37°N, (middle) 40°N, and (bottom) 42.5°N. (Left panels) For each city (*a* to *l*), a point represents a pair (${T}_{break},\theta$) for one of the 365 days of 2007. The scatter plots show that *T*_*break*_ behaves differently for different temperature ranges (blue points correspond to “cold” or “cool” days and red points to “warm” or“hot” days), separated by a threshold temperature θ* below which *T*_*break*_ seems to stay more or less constant and above it increasing with temperature. (Right panel) For each of the three latitudinal bands variabilities of: the threshold temperature θ* (first column) and the correlation *r* between *T*_*break*_ and *θ* when the temperature is below (second column) and above (third column) the threshold temperature θ*. (The color labeling (green, orange, indigo or brown) of cities in each latitudinal band is the same in both panels). The correlation are calculated using points above the temperature thresholds, turning out to be strong in all cases with values >99%. For temperatures below the threshold, the correlation is weak with *p*-values in general greater than 0.05. The points were clustered as below (blue) and above (red) the threshold by using a spectral clustering technique based on the Shi-Malik algorithm^55, 56^.

In order to study the influence of the daily temperature on the dynamics of *T*_*break*_, we present it as a scatter plot with the maximum daily temperature *θ* for the 12 above mentioned cities, as depicted in Fig. 4. It can be seen that *T*_*break*_ follows two different dynamics, distinguishable by a threshold temperature θ*, below which *T*_*break*_ seems not to be strongly influenced by the temperature and above which there is a clear linear trend (see Fig. A5 in the SI for a comparison between the time-series). To make this distinction more visible and to estimate the threshold, we apply a clustering algorithm to split the data-points into two sets, each one containing those points representing the dynamics below or above the threshold temperature. As the scatter of the data points is not compact and they are not spread along two separate regions with a well defined border, the density-based clustering algorithms^57, 58^ are not well-suited for determining a turning point in the dynamics. Instead we chose to use a spectral clustering technique based on the Shi-Malik algorithm^55, 56^, where a graph is built from the data points and mapped into a low-dimensional eigen-space, where the graph is partitioned based on the similarities of the eigenvectors. We use an implementation of this algorithm found in a standard machine learning library^59^. This spectral clustering technique requires to set the number of clusters that data points must be split into. In order to search for the above-mentioned turning-point in the dynamics we set the number of clusters to two, which corresponds to the visual evidence (see Fig. 4) of the presence of two different behaviors of the afternoon resting period. An evaluation of different choice of parameters is shown in the SI (Figs. A8–A9). The results for each city can be seen in Fig. 4, in the form of two sets (red and blue points), with a threshold temperature ranging from 18° to 25° (Fig. 4). Once each set is split into two subsets, we calculate the correlation between *T*_*break*_ and *θ* for the set of cities (Fig. 4, right panels). We observe a weak correlation when the temperature is below the threshold, i.e. for almost all of the 12 cities *r* ≤ 0.25 and *p* ≈ 0.05. On the other hand, when the temperature is above the threshold, we observe the correlations to be strong, i.e. *r* ≥ 0.5 and *p* ≈ 0.0001, which indicates a strong influence of ambient temperature on the afternoon break period *T*_*break*_.

Here we have shown that both, the daily NPR and DPR (i.e. the nocturnal and diurnal resting periods), as well as the characteristic times *T*_*night*_ and *T*_*break*_ follow specific dynamics showing seasonal and geographical variations. As depicted in Fig. 2 these resting periods seem to follow opposite dynamics across the year such that when added up, the resulting total daily resting period shows only a small variation over the year (see Fig. A6 in the SI). This lends support to the notion that the total daily resting time of people is more or less conserved.

## Discussion

At the individual level the SWC is known to depend on various physiological and social factors that make it difficult to identify the precise causes influencing its dynamics. However, studying the yearly variation of the NPR averaged over a big population gives us quite unique insight into its complex behavior and helps us to identify some of the key influencing factors. The SWC is bounded inside the NPR and we expect that both entities follow similar dynamics but with somewhat different onset and termination times. We have shown that the duration of the NPR, characterized by the width *T*_*night*_, closely follows the seasonal variation in the duration of the night. Moreover, we have observed that the duration of NPR is influenced by the latitude of the city in question. The difference between *T*_*night*_ in winter and in summer for the people in cities located at 36°N was found to be almost twice as much as that for the people in cities located 8° further north. It seems that the activity of people in southern cities are most affected by the length of the night (or conversely length of the daylight), despite the fact that in northern locations the length of the night changes faster and spans a bigger time interval than more southern latitudes.

Human physiology and its hormonal regulation is expected to govern the dynamics of the SWC at the individual level. In particular, the melatonin hormone is known to follow a circadian rhythm^60^ and has been linked to the SWC. For individuals, melatonin secretion commences during the evening between 8 p.m. and 10 p.m., peaking between 2:00 a.m. and 4:00 a.m. However, the onset and the duration of melatonin secretion during the circadian cycle is in turn related with the exposure to light and darkness, and considerable research has been done^61–64^ showing how changes in the onset and length of the light exposure disrupts the secretion of melatonin, as well as the SWC^65^. For people living at latitudes far away from the equator, where the seasonal changes in the onset and length of daylight are large, it has been shown that their melatonin cycle is seasonally altered and this perturbation gives rise to disruptions in their SWC^66^. Thus it could be possible that the melatonin cycle is participating in the entrainment of the SWC (and the NPR) with the duration of the daylight across the year. On the other hand, the diurnal period of resting DPR is found to depend rather strongly on the temperature, making its duration during hot days longer, but showing no noticeable variation during cold or cool days. Surprisingly, however, this increase in the daily diurnal resting time is found to be counterbalanced by the decrease of the daily nocturnal resting time. Hence it seems that on warm or hot days a bigger fraction of the total resting period is taken by the afternoon break, thereby reducing the homeostatic pressure for sleeping and consequently the need for nocturnal resting period.

The evidence of the trade-off between the afternoon and nighttime rest or sleep at latitudes closer to the tropics has implications for our understanding of the time budgets of tropical hunter-gatherers who have also been reported to take afternoon ‘naps’ for about a quarter of a day^20^. Our results suggest that, in high temperatures tropical habitats, humans find it difficult to remain active during the afternoon when ambient temperatures are at their highest, much as in the case of for most monkeys and apes^67, 68^. Figure 4 suggests that there is a critical threshold at which rest becomes necessary and that this occurs at ambient temperatures somewhere between 20–25 °C (though ‘naps’ of significant length are probably not common until ambient temperatures exceed ≈30°). This has important implications in that it removes a significant amount of time from the active working day, thereby reducing the time that can be devoted to ecologically or economically more important activities. Such constraints have imposed significant limitations on our species’ ability to evolve into complex societies^69^.

## Methods

### Time binning and smoothing of distributions

To generate each probability distributions, we divide the temporal axis in 5 minutes bins, in such a way that each distribution *P*_*all*_ contains 268 points, whilst *P*_*L*_ and *P*_*F*_ contain 132 points each one. In some cases, mainly for cities with small population, their associated probability distributions show fluctuations due to small sample size, then we apply a smoothing process to each distribution in order to reduce the noise, using the Savitsky-Golay^70^ algorithm (7 points and degree 4).

### Data filtering

From our dataset, two locations could be associated to each user. First one is the location of the most accessed cell tower (MACT) by the mobile device. From the zip code of the domicile of the user we get the second one, namely the location of the centre of the postal zone (postal location). These two locations were used when determining which users should be included in the analysis. We discarded those users with at least one of these two locations missing.

To reduce the noise due to poor sampling, we chose from the whole set of towns included in the mobile phone network only those cities with more than hundred thousand inhabitants in the studied year, and lying between latitudes 36°N and 44°N, which accounts for 36 cities. For each selected city, we encircled it with a 30 km diameter circle, and use the center of the circle as the associated geographical location of the city. All the studied cities fit inside a 30 km diameter circle.

Finally, we choose only those users who live in one of these cities. To ensure this into some extent, we impose the next two restrictions:

- The distance between the city location and at least one of the associated user locations (MACT and postal) should be less that 15 km, and
- The distance between the MACT and postal locations of the user must be less than 30 km.

There is no way to verify that the user actually lives in the associated city, but one could expect that almost every user lives in the same city as the one specified by the domicile, and that the MACT is indicative of their usual location. Thus the imposed restrictions seem to be a good way to ensure that a big fraction of the included users are correctly assigned to their actual city.

From the demographic information available, one can find the age of the users ranges from eighteen to more than hundred years. In this work we choose only users with age between 30 and 75 years old, because the calling pattern in younger people (mainly in the 18 to 25 years range) is more erratic than for older people. Older people (75+) were excluded the analysis due to the sparse calling activity that many of them had in the CDRs. After the previous filtering, the included users into the analysis were 925, 135 individuals.

### Cross-Correlation

To measure the possible time delay between the time-series ${T}_{night}(d)$ and ${N}_{length}(d)$ we calculate the cross-correlation $\rho \equiv \rho (\tau )$ between them, finding the time delay at which the cross-correlation reaches its maximum, meaning that the time-series are in the best possible alignment (in phase).

The (normalized) cross-correlation $\rho \equiv {\rho }_{XY}(\tau )$ for a time delay *τ* between two time-series $X(t)$, $Y(t)$, is given by

1 $${\rho }_{XY}(\tau )=\frac{1}{{\sigma }_{X}{\sigma }_{Y}}{\rm{E}}[(X(t)-\bar{X})(Y(t+\tau )-\bar{Y})],$$

where E[] denotes the expectation value, and $\overline{X}$, $\overline{Y}$ and *σ*_*X*_, *σ*_*Y*_, are the average values and the standard deviations of *X* and *Y*, respectively.

Using the discrete form of $X\equiv \{{X}_{0}\mathrm{..}{X}_{n-1}\}$, $Y\equiv \{{Y}_{0}\mathrm{..}{Y}_{n-1}\}$, with *n* the number of samples, and time delay *τ* as an integer between −*n*/2 and *n*/2, then the cross-correlation reads as follows

2 $${\rho }_{XY}(\tau )=\frac{1}{n-\tau }\sum _{i=max(0,\tau )}^{min(n-1+\tau )}(\frac{{X}_{i}-\bar{X}}{{\sigma }_{X}})(\frac{{Y}_{i+\tau }-\bar{Y}}{{\sigma }_{Y}}),$$

where $\overline{X}=\sum {X}_{i}/n$, $\overline{Y}=\sum {Y}_{i}/n$, ${\sigma }_{X}={(\overline{{X}^{2}}-{\overline{X}}^{2})}^{0.5}$, and ${\sigma }_{Y}={(\overline{{Y}^{2}}-{\overline{Y}}^{2})}^{0.5}$, the average and standard deviation of *X* and *Y*, calculated accordingly the usual definitions. For a given *τ*, the lower and upper bound of the summation as well as the denominator (n-*τ*) are chosen in such a way that only the overlapping part of the time-series is contributing to calculation of the cross-correlation.

### Data availability

The datasets analysed during the current study are not publicly available due to a signed non-disclosure agreement. The dataset contains sensitive information of the subscribers, particularly age, gender, postal address and the location of the most accessed cell tower. Calling time distributions from which all the results shown in this work were generated are available from the last author on reasonable request.

## Electronic supplementary material

Supplementary Information
