# Supplementary material for: Seasonal and geographical impact on human resting periods
Source: Sci Rep. 2017 Sep 6;7:10717. doi: 10.1038/s41598-017-11125-z (PMC5587566; doi:10.1038/s41598-017-11125-z)
Supplement: Supplementary file 1 — Supplementary Information [file 41598_2017_11125_MOESM1_ESM.pdf]

Supplementary Information of “Seasonal and geographical impact on human resting periods”

Daniel Monsivais[1,*], Kunal Bhattacharya[1], Asim Ghosh[1], Robin I. M. Dunbar[2,1], and Kimmo Kaski[1,2]

[1]Department of Computer Science, Aalto University School of Science, Espoo, P.O. Box 15400, FI-00076 AALTO, Finland
[2]Department of Experimental Psychology, University of Oxford, South Parks Rd, Oxford, OX1 3UD, United Kingdom
[*]daniel.monsivais-velazquez@aalto.fi

Time-series filtering due to holidays and atypical days filtering, and mean times for the first and last call

When calculating the probability distributions from the CDRs, we found that for some particular days and cities, the behavior of these distributions were atypical. The corresponding days when these anomalies appeared were in general special days, when some festivity or holiday was held, mainly Easter holidays (days 90 to 98) and the days around Christmas and New Year. In order to avoid possible fluctuations introduced by these atypical days, we excluded these days from the analysis. When there was a national holiday, the point corresponding to that day was removed from the time-series of all cities, and if the atypical day was a local festival or local event, that day was removed only for that city. Examples of this filtering can be seen in Fig. A1.

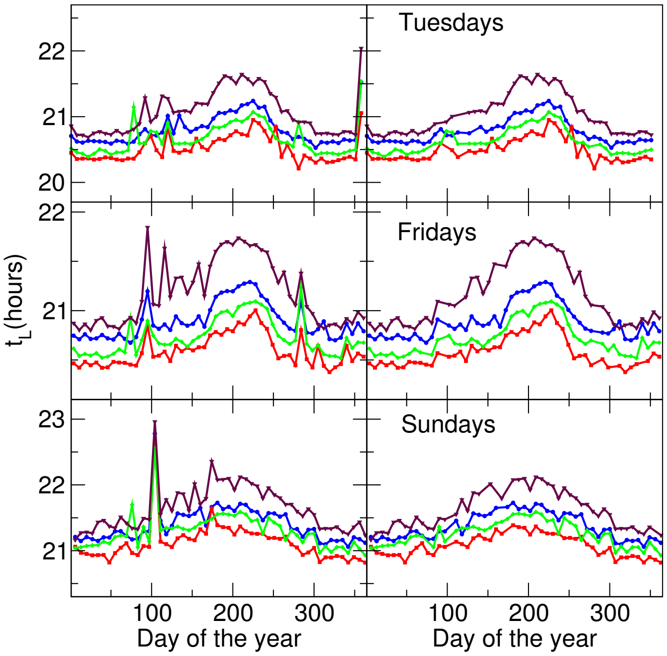

Figure A1. Removal of days when a national holiday, local festival or special event is present for the most 4 populated cities. (left column) Original t_L for each city. (right column) Filtered t_L.

In Fig. A2 and Fig. A3 the mean times for the first call $\bar{t}_L$ and for the last call $\bar{t}_F$ are shown, respectively, for 12 cities located along three different latitudes. To enhance the visualization of the time-series in each band so as to emphasize the similarity in shape, each time-series is vertically shifted by an amount proportional to the time difference between the local sun transit time of each city and the corresponding time at some reference point in the middle of the band.

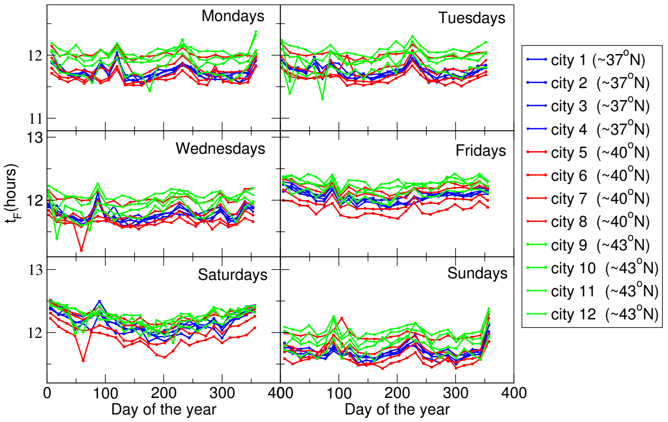

Figure A2. Mean time between of the first call $\bar{t}_F$ for 12 different cities during one year. The cities are grouped in 3 sets with 4 cities per set. Each set is located along one of these three latitudes $37°N$, $40°N$, or $43°N$. The time when the first call is made slightly changes along the year, specially during the weekends. Also southern cities start the calling activity slightly earlier. The peaks shown in the graphs correspond to certain special days, like holidays and festivals. Each time-series is vertically shifted by an amount proportional to the time difference between the local sun transit time of each city and the corresponding time at some reference point in the middle of the band, to enhanced the visualization of the time-series in each band, which show similar shapes.

Linear regression of T_{night} on N_{length}

From the linear regression, $\overline{T}_{night} = \beta \overline{N}_{length} + \alpha$, used quantify the yearly variation of $\overline{T}_{night}$ as a function of $\overline{N}_{length}$, in subsection "Influence of latitude in seasonal variability of low-activity period" in the main text, only δ_{season} was shown. Here we present the corresponding plot of the slopes β of the linear regression. Again, $\overline{T}_{night}$ is defined as the average value of T_{night} from Monday to Thursday each week, to characterize typical weekdays and to reduce the fluctuations of T_{night}.

Ambient temperature and afternoon break period time-series

Ambient temperature θ series during 2007 were obtained from the databases available from the national meteorological institute of the country where the cities are located. The available information contains the daily minimum, mean and maximum ambient temperatures on one of the monitoring station located in each city. For some cities, the information for some day was not present in the dataset, and the missing data was interpolated after applying a smoothing process (Savitzky-Golay) to the time-series. We compare θ and afternoon break period T_{break} for 12 cities, located along one of these three latitudes $37°N$, $40°N$, or $43°N$, and a there is a strong similarity between their behavior during warmer months (April - October generally). From the beginning of that period, θ and T_{break} increase monotonically, reaching their maximum value around August and then monotonically decrease until beginning of Autumn. Outside that period (colder months), θ follows its seasonal variation, but T_{break} stagnates, and shows no relation with the ambient temperature, as can be seen in A5.

Total daily resting period

The total period of low activity or of resting defined as $T_{rest} = T_{break} + T_{night}$ is calculated. T_{rest} is the consequence of two competing processes, the afternoon or diurnal resting period T_{break} and the night or nocturnal resting period T_{night}. Despite the seasonal variation of these two processes, the yearly changes T_{rest} are smaller and, particularly during the weekends appears to be uniform.

Estimation of the fraction of users active during afternoon break

Despite the noticeable reduction of the calling activity during the afternoon period on any given day, there is a set of users calling during that time. This prevents the calling activity from falling to zero unlike in the case of the night resting period. There are multiple reasons for this background activity. For example, some users take an afternoon break in their routine activities before or after the characteristic time of the population as a whole, while some individuals simply do not take this break. We make a gross estimation of the fraction of inactive users at that time in the following fashion. We focus on subset of 'active' users, including in it only those who have at least a certain number of calls during the day and making their calls in both halves of the day, namely, the morning and the evening periods. With these criteria, we can expect that the included users have enough calls that are spread over the entire span of the day. Once we have determined this set of users, we divided the day into

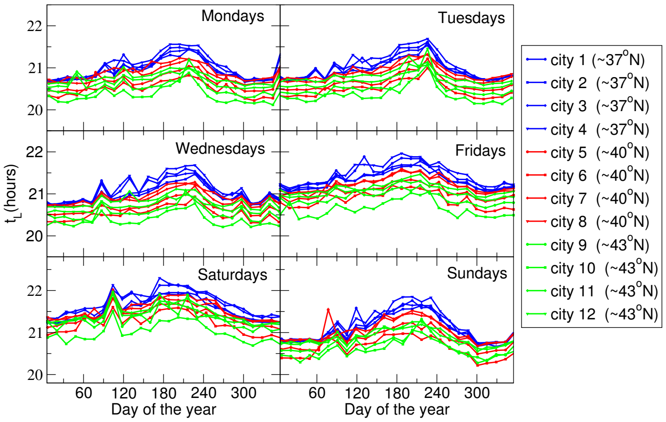

Figure A3. Mean time of the last call $\bar{t}_L$ for 12 different cities during one year. The cities are grouped in 3 sets with 4 cities per set. Each set is located along one of these three latitudes $37°N$, $40°N$, or $43°N$. The time when the last call changes strongly along the year, specially during the weekends. Southern cities cease the calling activity later, and the size of the seasonal change is stronger than in the case of northern cities (the time difference between summer and winter days is bigger for southern cities). The peaks shown in the graphs correspond to certain special days, like holidays and festivals. Each time-series is vertically shifted by an amount proportional to the time difference between the local sun transit time of each city and the corresponding time at some reference point in the middle of the band, to enhanced the visualization of the time-series in each band, which show similar shapes.

three different time periods between 4:00 am to 2:00 p.m., 2:00 p.m. to 5:00 p.m. (approximately coinciding with the T_{break} period), and 5:00 p.m. to 4:00 am (of the next calendar day), respectively.

To estimate the fraction of users resting during T_{break} period, we include those users with 3 or more calls over the day (with other restrictions as stated above), and calculate the fraction of users that don't make calls during the T_{break} period. This coarse estimation gives us insight of how active users behave along the day, and allows us to determine how many of them reduce their activity during the afternoon break. The subset of users are at best a sample of the population, but at least represent the overall behavior. The results for the four most populated cities are shown in Fig. A7 and it can be seen that the fraction of inactive users during the afternoon break is around 0.5 across the year.

Parameters estimation for the clustering algorithm

We compared the resulting partition of the data for different numbers of clusters (see Fig. A8), and the case for 2 clusters gives a good partitions (when 4 clusters were imposed, the algorithm gave a partition equivalent to the case of 2 clusters, dividing each set into two parts). Another parameter required by the algorithm is related with the similarity graph, which is built from the k nearest neighbors of each node. We evaluated different values (see Fig. A9) and choose $k = 10$ to build the graph.

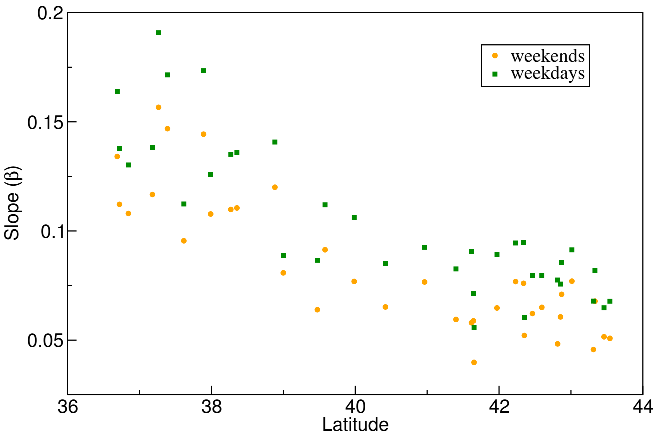

Figure A4. Latitudinal dependence of the net change δ_{season} between $\overline{T}_{night}$ at winter solstice (maximum) and at summer solstices. Coefficient of linear regression, β, obtained for each of the 36 studied cities as a function of its latitude. In southern cities, the width of the NPR changes faster (larger βs) and the difference between their minimum (summer solstice) and maximum (winter solstice) values is larger than in case of the cities in the north. Weekdays points (blue) were calculated from the average $\overline{T}_{night}$ from Mondays to Thursdays. For weekends points (red), Friday to Sunday were used to calculate the average $\overline{T}_{night}$.

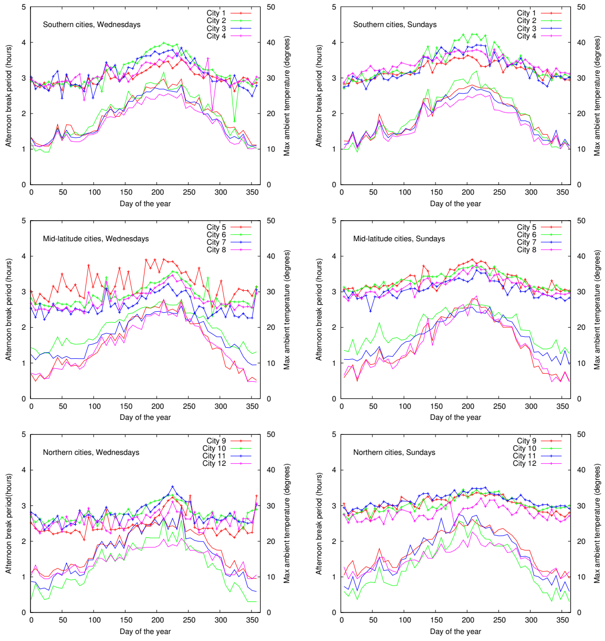

Figure A5. Afternoon break period yearly dynamics compared with the corresponding temperature time-series, for 4 different cities lying at different 37°N, 40°N and 42.5°N latitude. The lines with markers represent the length of the afternoon break resting along the year, whilst those without markers represent the max temperature in those cities. Each city is represented by the same color in both time-series. Wednesdays and Sundays are shown

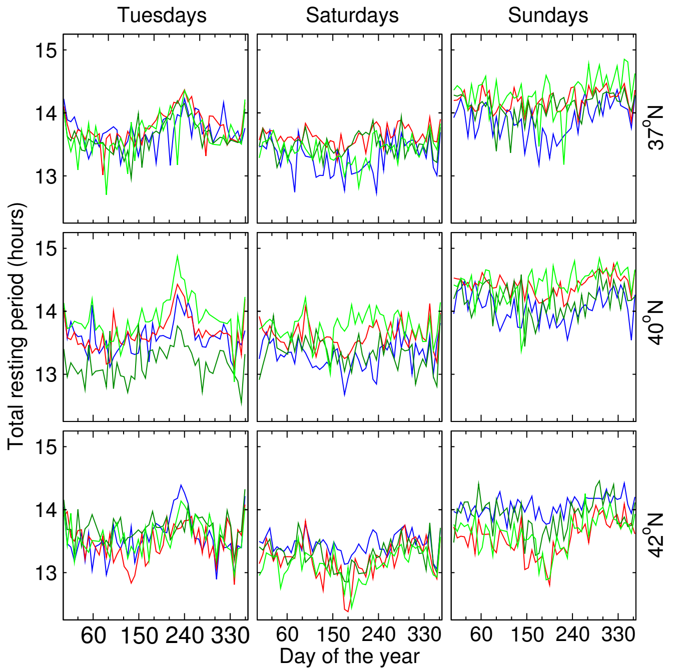

Figure A6. Total period of low calling activity. The total period of low activity or of resting defined as the sum of T_{break} and T_{night} for 12 different cities across 2007 for 3 different days of the week. The cities are located in one of the three different latitudinal bands (top) 37°N,(middle) 40°N, and (bottom) 42.5°N. T_{rest} is the consequence of two competing processes, the afternoon or diurnal resting period T_{break} and the night or nocturnal resting period T_{night}. Despite the seasonal variation of these two processes, T_{rest} is almost uniform across the year, particularly during the weekends.

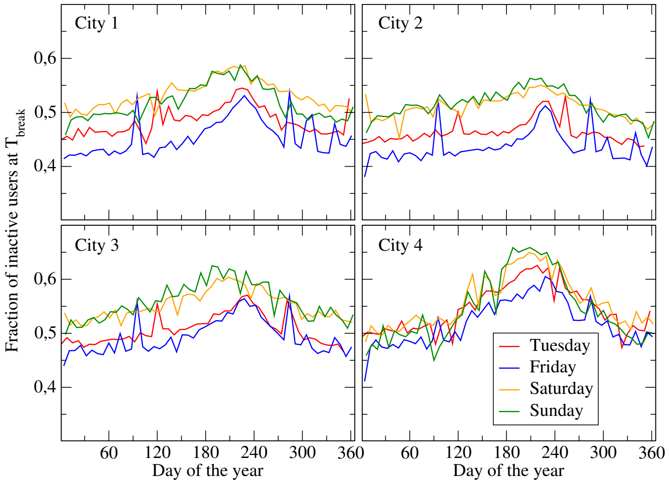

Figure A7. Fraction of inactive users during afternoon resting period. The set of users used in this analysis made more than three calls that day, at least one before and one after 3:30 pm. From these users, we calculate the fraction of them that were inactive during the period 2:00pm - 5:00 pm. The fraction was calculated for the four most populated cities across 2007 for 4 different days of the week. Cities 1, 2, 3 and 4 are located around latitudes 40°N, 42°N, , 39°N, and 37°N, respectively.

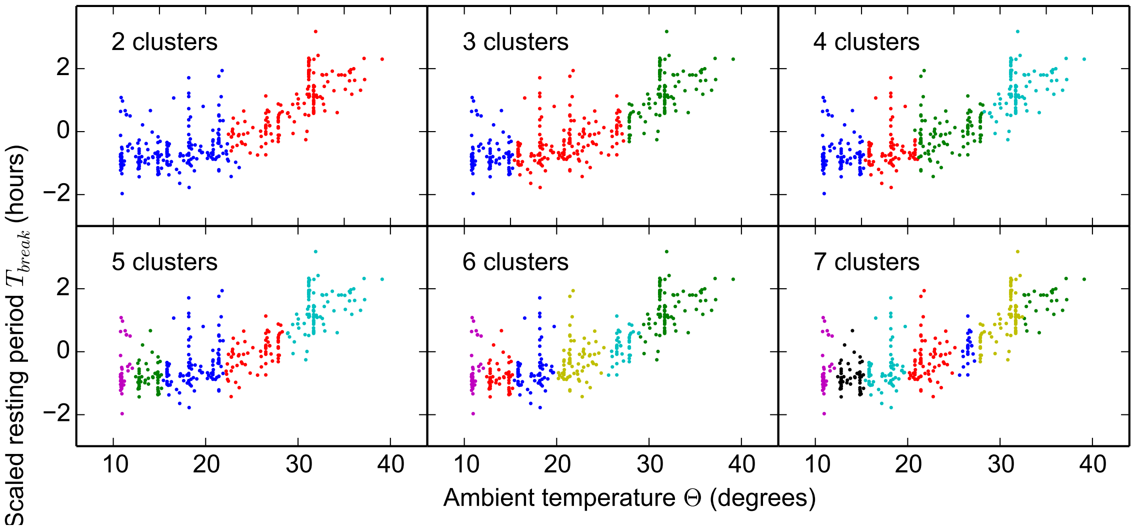

Figure A8. Comparison of the resulting clusters for different values of the k-nearest neighbors parameter, for one of the analyzed cities. For $k >= 6$ the clustering algorithm gives a consistent partition of the data, with a threshold temperature Θ^* around 28°. For all the plots, the number of clusters was set to 2.

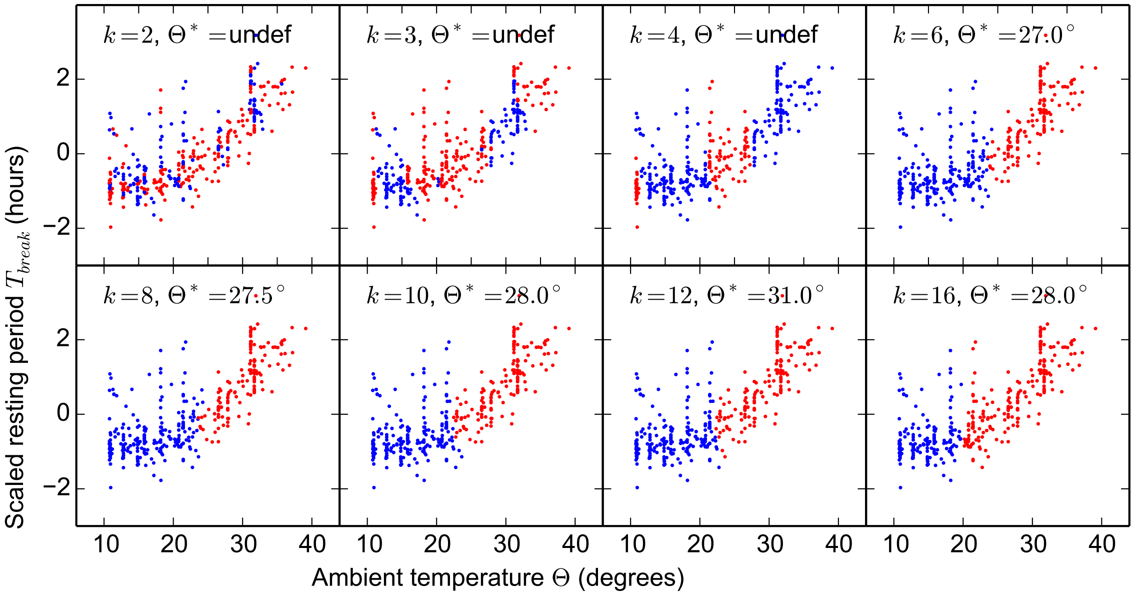

Figure A9. Comparison of the resulting partition of the graph between different number of clusters, for one of the analyzed cities. For the case of two clusters, the partition of the points shows clearly the region where T_{break} changes its behaviour. For the case of three clusters, the temperature at which T_{break} changes its behaviour falls inside the central cluster, therefore it is not a good choice. In the case when 4 clusters was chosen, the result is similar than the case when 2 clusters were imposed. The first set (in red) for the 2 clusters case is split in two sets (in red and cyan) in the 4 clusters case. Similar situation with the second cluster (in red) for the 2 clusters case, being partitioned in two sets (in green and blue). In all cases, $k = 10$.
